# Extending access to a web-based mental health intervention: who wants more, what happens to use over time, and is it helpful? Results of a concealed, randomized controlled extension study

**DOI:** 10.1186/s12888-019-2030-x

**Published:** 2019-01-24

**Authors:** Jennifer M. Hensel, James Shaw, Noah M. Ivers, Laura Desveaux, Simone N. Vigod, Zachary Bouck, Nike Onabajo, Payal Agarwal, Geetha Mukerji, Rebecca Yang, Megan Nguyen, Lianne Jeffs, Trevor Jamieson, R. Sacha Bhatia

**Affiliations:** 1 Women’s College Hospital Institute for Health Systems Solutions and Virtual Care, Women’s College Hospital, 76 Grenville St, Toronto, Ontario M5S 1B2 Canada; 2 Department of Psychiatry, Women’s College Hospital and University of Toronto, Toronto, Ontario Canada; 3 Women’s College Research Institute, Toronto, Ontario Canada; 4 Department of Psychiatry, University of Manitoba, Winnipeg, Manitoba Canada; 5 Department of Family and Community Medicine, Women’s College Hospital and University of Toronto, Toronto, Ontario Canada; 6 Institute for Health Policy, Management and Evaluation, University of Toronto, Toronto, Ontario Canada; 7 Department of Medicine, University of Toronto, Toronto, Ontario Canada; 8 Li Ka Shing Knowledge Institute, St. Michael’s Hospital, Toronto, Ontario Canada

**Keywords:** Web-based, Internet, E-health, Peer support, Randomized controlled trial, Recovery, Implementation

## Abstract

**Background:**

Web-based mental health applications may be beneficial, but adoption is often low leaving optimal implementation and payment models unclear. This study examined which users were interested in extended access to a web-based application beyond an initial 3-month trial period and evaluated if an additional 3 months of access was beneficial.

**Methods:**

This study was a concealed extension of a multi-center, pragmatic randomized controlled trial that assessed the benefit of 3 months of access to the Big White Wall (BWW), an anonymous web-based moderated, multi-component mental health application offering self-directed activities and peer support. Trial participants were 16 years of age or older, recruited from hospital-affiliated mental health programs. Participants who received access to the intervention in the main trial and completed 3-month outcome assessments were offered participation. We compared those who were and were not interested in an extension of the intervention, and re-randomized consenting participants 1:1 to receive extended access or not over the subsequent 3 months. Use of the intervention was monitored in the extension group and outcomes were measured at 3 months after re-randomization in both groups. The primary outcome was mental health recovery as assessed by total score on the Recovery Assessment Scale (RAS-r), as in the main trial. Linear mixed models were used to examine the time by group interaction to assess for differences in responses over the 3-month extension study.

**Results:**

Of 233 main trial participants who responded, 119 (51.1%) indicated an interest in receiving extended BWW access. Those who were interested had significantly higher baseline anxiety symptoms compared to those who were not interested. Of the 119, 112 were re-randomized (55 to extended access, 57 to discontinuation). Only 21 of the 55 extended access participants (38.2%) used the intervention during the extension period. Change in RAS-r scores over time was not significantly different between groups (time by group, F(1,77) = 1.02; *P* = .31).

**Conclusions:**

Only half of eligible participants were interested in extended access to the intervention with decreasing use over time, and no evidence of added benefit. These findings have implications for implementation and payment models for this type of web-based mental health intervention.

**Trial registration:**

Clinicaltrials.gov NCT02896894. Registered retrospectively on September 12, 2016.

## Background

Mental health problems are a leading cause of disability with significant health and societal cost implications [1]. Web-based applications offering mental health interventions are emerging as flexible solutions to barriers in access to care [2]. These interventions have garnered some evidence for various mental health needs and across a range of intervention components, with higher engagement often associated with better outcomes [3]. However, sustained engagement with web-based health interventions has repeatedly been identified as a limitation of their general utility, not only in mental health populations [4–6].

A recent review of digital mental health applications for depression and anxiety found the rate of sustained use of the applications beyond 6 weeks or more to range from a low of 0.5% to just under 30%, with the highest proportion of users engaging in very brief use [7]. What was notable in these studies was the fact that some applications achieved very low adoption overall, but among adopters there were some who were persistently high users [7]. Further complicating matters is that actual use of web-based platforms can be measured in a variety of ways, most commonly defined as number of logins to the portal [3, 5]. Some reports, however, have described users who login but do not participate actively in the program components – such as not performing recommended tasks in modular-style programs, or not posting any content in peer communication forums [8, 9]. This makes the determination of adequate usage additionally challenging to quantify and correlate with outcomes, particularly for multi-component interventions that don’t necessarily have a defined dose or target level of engagement [6]. Moreover, many web-based platforms work on a license- or subscription-based payment model, so low engagement by users has implications for payors, such as public or private insurers, that purchase these programs. For example, an expensive intervention, assuming it is beneficial, that garners few engaged users will not yield a high value for money and will necessitate significant investment in user acquisition, relative to a less expensive intervention that appeals to a large audience of users.

The Big White Wall (BWW) [10] is an example of a subscription-based, multi-component, moderated web-based intervention with peer support and self-directed modular activities hosted in a virtual environment that provides anonymity. The content offered by this intervention is not specific to any particular mental health diagnosis and may thereby be beneficial to a wide base of users with various mental health needs. Our group undertook a large, multi-site randomized controlled trial of the BWW in Ontario (Canada’s most populous province) to compare three months of BWW access to a control group who received access after a 3-month waiting period [11]. The study reported herein was an extension of that trial that aimed to characterize those interested in longer-term access to the BWW and their use over time, as well as to evaluate whether extended access to BWW (i.e. a total of 6 months of access) was beneficial compared to discontinued access (i.e. 3 months of access) with respect to scores on a mental health recovery measure. Specifically, the study had three research questions:

1. What proportion of intervention users after 3 months were interested in extended access to the same intervention for another 3 months, and how do they compare with users who were not interested?
2. Among users with extended access, what does usage look like in the second 3 months of access and how does it compare to usage in the first 3 months?
3. Among those interested in extending access to the intervention, is there benefit of extended access compared to a group who has their access discontinued?

An overarching objective of this study was to assist clinicians and policymakers in determining the optimal timing of access to the intervention that would inform both payment models for the technology and implementation approaches for wider scale adoption of this or similar interventions.

## Methods

### Study design

This study was an extension of a multi-center, parallel-arm, pragmatic randomized controlled trial of the BWW among participants seeking services at specialized mental health and addictions programs at the participating sites. The main trial compared 3 months of access to the intervention to a delayed access control group. The BWW developer offered a default 6-month licensing model to the study sponsor, however, we requested a shorter exposure period for the main trial because we anticipated low uptake of the intervention based on literature describing this pattern [3–5]. We chose 3 months since it represented half of the duration of the default license and the developer was willing to offer it, and it aligned with the typical duration of brief psychological treatments shown to be effective [12].

At the 3-month endpoint of the main trial, participants completing the outcome measures were invited to opt into this extension study, which offered the opportunity to be re-randomized to an additional three months of intervention access or to have intervention access end as planned. At the time of enrollment into the main trial, participants were told that they would be required to complete follow-up surveys at both 3 and 6 months, but the extension study was concealed at the outset to encourage maximal use of the intervention during the initial 3-month exposure period. Participants who opted in to the extension study were re-randomized 1:1 to receive an additional 3 months of the intervention (extension group) or no additional access to the intervention (discontinuation group), in which case their access to the intervention ended. This extension study was included in the trial protocol, which has been published [11].

### Recruitment of participants

Participants for this study represent a subsample of the participants in the main trial. All participants in the main trial were actively seeking and/or receiving treatment from outpatient mental health programs affiliated with three participating sites in Ontario, Canada. The sites consisted of a general hospital with a large mental health program (Site A), a specialized provincial mental health facility (Site B), and an ambulatory care hospital focused on women’s health but not exclusively serving a female population (Site C). To recruit an intentionally heterogeneous population given the non-specific diagnostic focus of the intervention and to meet sponsor recruitment targets, we recruited from a range of outpatient programs: mood and anxiety psychiatry and psychotherapy programs (both adult and youth), an urgent care program and an emergency department (at time of discharge), trauma therapy programs, and a substance use program.

Participants who completed the outcome assessment at the 3-month end-point of the main trial received automated questions within the web-based follow-up survey asking them if they were interested in an opportunity for extended access to the BWW, and if they responded yes, if they consented to re-randomization to receive extended access or not (completely optional), which would provide them with an opportunity to receive an additional three months of access to the intervention. Consistent with the main trial, all participants in this extension study were 16 years of age or older, had access to the internet and an email address, were able to read English, and willing and able to access and use an online mental health intervention. In this extension study, as in the main trial, there were no restrictions on the use of concomitant care, including accessing other web-based interventions akin to the study intervention. All participants provided informed consent. All outcome data were collected into a REDCap database [13]. The main trial began in July 2016 with recruitment into this extension study occurring between October 2016 and April 2017.

### Intervention – The big White Wall™

The BWW is an anonymous, moderated, multi-component web-based mental health application operated from the United Kingdom [10]. In the main trial, all participants allocated to the intervention group were given immediate access to the BWW for three months, free of charge. In this extension study, those who were randomized to an extension, received an additional three months of access free of charge. The same user accounts were reactivated for extended users to ensure continuous access to the same user profiles. A main component of the BWW is its moderated, peer support platform. The platform is monitored 24/7 by ‘Wall Guides’, trained mental health professionals under the supervision of clinical psychologists and psychiatrists, who constantly monitor activity and can engage in communication with users if needed. For example, Wall Guides will respond to user posts that go unanswered in the peer community. The BWW also includes a range of self-directed components including educational pages (referred to as Useful Stuff pages), guided support courses that cover a range of mental health-related topics (eg. grief, depression, anxiety, smoking cessation, trauma, among others), and artistic creations referred to as ‘bricks’ that get posted to the peer forum. If there has been a prolonged period of inactivity, users receive a notification through their registration email encouraging them to log on. For our study, all participants received an email alias that linked to their personal email and a unique prescription for a BWW account. Technical support throughout the study was available from the research team, the BWW, and the Ontario Telemedicine Network.

### Outcomes

We obtained actual BWW utilization data for all extension participants, both during their first 3 months of access as part of the main trial, and during the subsequent 3-month extension period for those receiving extended access. Use of web-based platforms is most commonly reported as number of logins to the portal, but a range of use measures can be examined [5]. Utilization data collected for this study included total number of logins, total time on the site in minutes, and number of activities including “talkabouts” or postings with peers and WallGuides, bricks, and support courses accessed.

We assessed the same outcomes as in the main trial at the end of the 3-month extension period to determine if there was benefit from the additional 3 months of access to the intervention compared to having access discontinued. As in the main trial, the primary outcome was mental health recovery assessed with the Recovery Assessment Scale-revised (RAS-r), a 24-item scale that reflects the “recovery era” for mental health policy and services where the focus is finding satisfying and fulfilling lives, rather than being entirely symptom free [14]. Secondary outcomes were symptoms of depression measured with the Patient Health Questionnaire-9 item (PHQ-9), symptoms of anxiety measured with the Generalized Anxiety Disorder Questionnaire-7 item (GAD-7), quality of life (QOL) measured with the Visual Analog Scale (VAS) out of 100 from EuroQOL group, [15] and community integration assessed with the 15-item Community Integration Questionnaire (CIQ) [16]. Higher scores on the PHQ-9 and GAD-7 scales represent more symptoms [17]. The VAS assesses perceived overall health at the time of survey completion. The CIQ consists of 15 items and is intended as a brief, reliable measure of a person’s level of integration into the home and community, with a higher score indicating better integration [18].

### Randomization, concealment, and blinding

Randomization sequences were computer generated by an organization external to the research team, with stratification by recruitment site – Site A (general hospital), Site B (provincial mental health facility), Site C (ambulatory care hospital). Block sizes of 2 or 4 were used for 1:1 randomization. Group allocation sequence was concealed but once allocated, participants were not blinded. Data collectors were blinded to group allocation throughout by rotating assessors if required.

### Follow-up

Follow-up data for this extension study were collected between January 2017 and June 2017. The primary outcome endpoint was 3 months post re-randomization. All follow-up data were collected by self-report via electronic surveys through REDCap or collected by phone or in-person by a study team member and subsequently input into the REDCap database. Participants received an automated survey link by email one week prior to 3-months, with a personalized email following within two days. If the survey was not completed by 3 months, the link was re-sent, and a reminder phone call was made by a study coordinator or research assistant. This was repeated at 3 months plus one week. Surveys were closed two weeks after the 3-month time point. Outcome measures were completed via the web-based survey in 95% of cases.

### Statistical analysis

We compared those eligible participants who expressed interest in extended access to the intervention to the participants who did not on baseline characteristics, baseline outcome measures, and prior 3-month BWW utilization defined as number of logins, with t-tests (or Mann-Whitney U tests in the event of skewness, e.g. total number of log-ins) for continuous variables, and chi-square tests of independence (or Fisher’s exact tests where appropriate) for categorical variables. Baseline for this comparison was considered to be the time of the offer for extended access.

Among those randomized to receive extended access to the intervention, BWW utilization data in the 3 months prior to the extension study was compared descriptively with use during the 3 months of extended access.

Multiple linear mixed effects regression was used to independently model data on primary and secondary outcomes over time among participants randomized to extended or discontinued access. We focused on the time by group interaction term in each linear regression model adjusted for fixed effects for time, group, and recruitment site with random intercepts to account for repeated, participant-level measures. Our linear mixed effects regression analysis assumed any missing outcome data at follow-up were missing completely at random. To test the sensitivity of our findings to this assumption, we identified whether any baseline characteristics were associated with the missingness or value (where observed) of the primary outcome. Any characteristics identified were then added as covariates to the main regression model and results were compared to those obtained from the main model assuming data were missing completely at random.

## Results

### Participant interest in extended access to the intervention

From 542 participants who received access to the intervention in the main trial, only 233 provided valid responses to the primary outcome survey at 3-months post-initial randomization and the invitation to opt-in to this extension study. Out of those, 119 (51.1%) indicated an interest in the opportunity for extended access to the BWW, with the other half saying no. The only baseline characteristics at the time of the offer for extended access that differed significantly between those who did and did not express interest in extended access was GAD-7 score (Table 1). Those with continued interest had significantly higher levels of anxiety at baseline (mean GAD-7 score of 9.8 for those interested vs 8.3 for those who were not, *P* < .05).

**Table 1:** Baseline characteristics for extension study participants by response to interest in extended access question

Baseline Characteristic	Interest in Extended Access		
	Yes *n* = 119	No *n* = 114	*P* value
Recruitment site, n(%)			.73
A (General Hospital)	56 (47)	57 (50)	
B (Provincial Mental Health Facility)	35 (29)	35 (31)	
C (Ambulatory Care Hospital)	28 (24)	22 (19)	
RAS-R total score, mean (SD)	83.0 (13.6)	83.5 (16.2)	.80
PHQ-9 total score, mean (SD)	12.1 (6.3)	10.7 (6.5)	.11
GAD-7 total score, mean (SD)	9.8 (5.2)	8.3 (5.4)	.04*
CIQ total score, mean (SD)	13.7 (4.7)	13.9 (5.2)	.81
EQ VAS, mean (SD)	57.1 (22.3)	61.6 (19.8)	.11
Total number of BWW log-ins in first 3 months, median (Q1,Q3)	7 (2,18)	6 (3,13)	.44
Age [y], mean (SD)	42.2 (12.4)	42.7 (14.3)	.82
Gender, n(%)			.73
Male	29 (24.4)	33 (28.9)	
Female	89 (74.8)	80 (70.2)	
Transgendered or other	1 (0.8)	1 (0.9)	
Ethnicity, n(%)			.34
White	98 (82.4)	99 (86.8)	
Non-white	21 (17.6)	15 (13.2)	
Relationship Status, n(%)			.58
In a relationship	69 (58.0)	62 (54.4)	
Not in a relationship	50 (42.0)	52 (45.6)	
Employment Status, n(%)			.89
Full-time (including homemaker with young children)	43 (36.1)	40 (35.0)	
Part-time/volunteer/homemaker without young children	18 (15.1)	20 (17.6)	
Not working	57 (47.9)	54 (47.4)	
Missing	1 (0.8)	0 (0.0)	
Household income [$CAD], n(%)			.70
< $35 K	47 (45.2)	38 (7.3)	
$35 K - $50 K	11 (9.2)	12 (10.5)	
$50 K - $80 K	20 (11.1)	21 (11.8)	
> $80 K	26 (26.5)	31 (31.6)	
Missing	15 (12.6)	12 (10.5)	
Age [y] first experienced mental health problems, mean (SD)	18.7 (12.1)	18.8 (12.3)	.96
Age [y] first sought help, mean (SD)	25.9 (12.5)	27.2 (12.9)	.44
Taking psychotropic medication, n(%)			.97
Yes	94 (79.0)	89 (78.1)	
No	25 (31.0)	24 (21.0)	
Missing	0 (0.0)	1 (0.9)	

Baseline represents the time point when extended access was offered. Percentages are column percentages and may not add exactly to 100.0% due to rounding. Statistical analyses exclude missing data

*SD* standard deviation, *Q1* first quartile, *Q3* third quartile, *CAD* Canadian: *RAS-r* Recovery Assessment Scale – revised, *PHQ-9* Patient Health Questionnaire – 9 item, *GAD-7* Generalized Anxiety Disorder Questionnaire – 7 item, *EQ-VAS* EuroQOL Visual Analog Scale (out of 100), *CIQ* Community Integration Questionnaire

*P < .05

### Outcomes of extended access

**Fig. 1:**
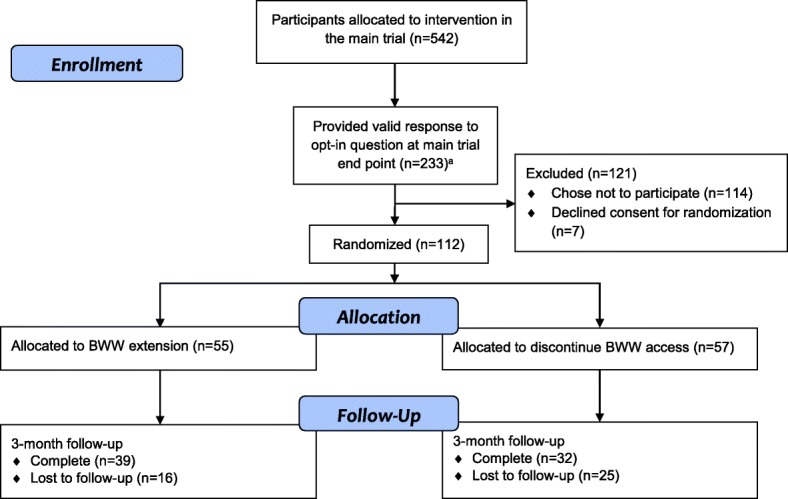
Flow of participants from the main trial through the extension study. ^a^6 additional participants provided a response deemed invalid because it was submitted beyond the required completion date and/or there was no primary outcome survey completed

Figure 1 details how the sample of 112 participants for the extension trial was arrived at from the main trial population. Among those expressing interest in extended access (*n* = 119), 7 did not provide consent to be re-randomized, leaving 112 who were re-randomized (55 to extended access, 57 to discontinuation). Responses for the primary outcome at 3-months after re-randomization were obtained for 63.3% of participants (*n* = 39 (70.9%) in the extension group and *n* = 32 (56.1%) in the discontinuation group). Baseline sociodemographic and mental health variables are presented by re-randomization group in Table 2.

**Table 2:** Baseline characteristics and first 3-month BWW utilization among extension trial participants

Baseline Variable	Extension Group *n* = 55	Discontinuation Group *n* = 57
Recruitment site, n(%)^a^		
A (General Hospital)	27 (47)	25 (45)
B (Provincial Mental Health Facility)	16 (28)	17 (31)
C (Ambulatory Care Hospital)	14 (25)	13 (24)
RAS-r total score, mean (SD)	82.4 (13.9)	83.8 (12.7)
PHQ-9 total score, mean (SD)	13.3 (6.6)	10.9 (5.7)
GAD-7 total score, mean (SD)	9.9 (5.0)	9.6 (5.4)
EQ VAS, mean (SD)	54.4 (24.3)	60.0 (19.6)
CIQ total score, mean (SD)	13.7 (4.7)	13.7 (4.4)
Total number of BWW log-ins in first 3 months, median (Q1,Q3)	7 (2, 22)	7 (2, 18)
Age, mean (SD)	40.6 (13.1)	43.0 (12.2)
Gender, n(%)		
Male	9 (16)	18 (32)
Female	46 (84)	38 (67)
Transgendered	0 (0)	1 (2)
Ethnicity, n(%)		
White	48 (87)	44 (77)
Non-white	7 (13)	13 (23)
Relationship Status, n(%)		
In a relationship	28 (51)	35 (61)
Not in a relationship	27 (49)	22 (39)
Employment Status, n(%)		
Full-time (including. Homemaker with young children)	14 (25)	26 (46)
Part-time/volunteer/homemaker without young children	11 (20)	6 (11)
Not working	29 (53)	25 (44)
Missing	1 (< 1)	0 (0)
Household income [$CAD], n(%)		
< $35 K	26 (47)	19 (33)
$35 K - $50 K	4 (7)	7 (12)
$50 K - $80 K	7 (13)	11 (19)
> $80 K	12 (22)	12 (21)
Missing	6 (11)	8 (14)
Age [y] first experienced mental health problems, median (Q1,Q3)	14 (8, 20)	16 (13, 29)
Age [y] first sought help, median (Q1,Q3)	19 (15, 27)	25 (18, 37)
Taking psychotropic medication, n(%)	48 (87)	40 (70)

Baseline represents the time point when extended access was offered. Percentages are column percentages and may not add exactly to 100.0% due to rounding

^a^Randomisation was stratified by recruitment site

*SD* standard deviation, *Q1* first quartile, *Q3* third quartile, *CAD* Canadian RAS-r: Recovery Assessment Scale – revised, *PHQ-9* Patient Health Questionnaire – 9 item, *GAD-7* Generalized Anxiety Disorder Questionnaire – 7 item, *EQ-VAS* EuroQOL Visual Analog Scale, *CIQ* Community Integration Questionnaire

### BWW utilization

Among the 55 participants who were randomized to receive an extension for the BWW, 21 (38.1%) used it at least once in the second three months. The median number of logins in the extension group declined from the first 3-month interval (median 7; interquartile range [IQR] 2 to 22) to the second 3-month interval (median, 0; IQR 0 to 2). Number of logins was the same or higher for only 7 participants in the extended group and decreased for all others. Utilization of all intervention components significantly decreased in the second three months (see Table 3).

**Table 3:** Use of intervention components during the first second 3 months of access among those offered extended access only (n = 55)

Component	First 3 months of access	Second 3 months of access
Total Logins, median (Q1,Q3)	7 (2,22)	0 (0,2)
Total time on site in minutes, median (Q1,Q3)	97 (28,295)	0 (0,22)
‘Useful Stuff’ educational pages, median (Q1,Q3)	103 (17,249)	0 (0,32)
Forum posts^a^, median (Q1,Q3)	3 (0,12)	0 (0,1)
Total number of support course sessions, median (Q1,Q3)^b^	0 (0,3)	0 (0,0)

^a^Includes interactions with peers and WallGuides, as well as creative ‘bricks’ and related posts

^b^Total number of sessions completed; individual courses consist of multiple sessions, so this may represent sessions completed in multiple courses

*Q1* first quartile; *Q3* third quartile

### Primary and secondary outcomes

The interaction between time and re-randomization group was not significant for RAS-r (*F*(1,77) = 1.02, *P* = .31) or any of the secondary outcomes: PHQ-9 (*F*(1,77) = 2.76, *P* = .10), GAD-7 (*F*(1,79) = 1.17, *P* = .28), EQ-VAS (*F*(1,76) = 0.52, *P* = .47) or CIQ (*F*(1,69) = 0.00, *P* = .99) (Table 4).

**Table 4:** Results of linear mixed effects models for primary and secondary outcomes after 3 months of extended Big White Wall access

Outcome	Extension Group, LS mean (SE) (n = 55)				Discontinuation Group, LS mean (SE) (*n* = 57)				Interaction term (time by group)			*P*-value
	n^a^	Baseline^b^	n^a^	3 months	n^a^	Baseline^b^	n^a^	3 months	v	d	F	
Primary Outcome												
RAS-r	55	82.0 (1.90)	39	82.0 (2.06)	57	83.3 (1.87)	32	81.0 (2.16)	1	77	1.02	.31
Secondary Outcomes												
PHQ-9	55	13.5 (0.86)	36	12.0 (0.97)	57	11.2 (0.85)	32	11.6 (1.0)	1	77	2.76	.10
GAD-7	55	10.0 (0.73)	36	9.2 (0.84)	57	9.8 (0.72)	32	10.2 (0.88)	1	79	1.17	.28
EQ-VAS	55	53.7 (3.0)	36	57.6 (3.6)	57	59.2 (3.0)	30	59.2 (3.9)	1	76	0.52	.47
CIQ	55	13.7 (0.62)	36	14.1 (0.69)	57	13.7 (0.63)	30	14.0 (0.73)	1	69	0.00	.99

All models were adjusted for fixed effects of group, time, and recruitment site with random, subject-specific intercepts to account for repeated measures

*LS mean* Least squares mean estimated from linear mixed effects models, Numerator (v) and denominator (d) degrees of freedom corresponding with F test statistic*, RAS-r* Recovery Assessment Scale – revised, *PHQ-9* Patient Health Questionnaire – 9 item, *GAD-7* Generalized Anxiety Disorder Questionnaire – 7 item, *EQ-VAS* EuroQOL Visual Analog Scale, *CIQ* Community Integration Questionnaire

^a^After missing data

^b^Represents baseline for the extension trial, which was at the time of re-randomization after the first 3 months of intervention access during the main trial

In our exploratory analysis to determine the impact of missing data, we found that the only factor associated with missingness was whether the individual was taking medication at baseline. After adjusting for medication use at baseline, the findings of our sensitivity analysis (time by group interaction, *F*(1,76) = 0.77, *P* = .38) did not differ substantially from our main results.

## Discussion

In this study, by concealing the opportunity to have extended access to the BWW intervention after an initial exposure period, we revealed some interesting findings about intervention users. Since uptake of the intervention in the main trial was low, we isolated a subset of trial participants who continued to be interested in the intervention at the main study 3-month endpoint. This represented less than a quarter of the initial study population, and only half of those responding to the question regarding interest in extended access. The only baseline variable that differed significantly between those expressing continued interest versus not was severity of self-rated anxiety symptoms. Of particular interest was that during the 3-month extension period, over 60% of eligible participants did not access the intervention at all. Use of all components decreased on average, but there was a very small group of users who demonstrated persistent regular use of the application. Overall, there was no statistically significant benefit observed for extended access beyond the initial 3-month period. This study yields important information about user behavior that could be beneficial to inform payment and implementation approaches for this and similar types of interventions.

To our knowledge, this is the first study to conceal an extension study and re-randomize interested users to receive extended access to a web-based intervention. The heterogeneous population we studied, recruited from specialized mental health services, likely represents more treatment-refractory individuals than studies done in primary care or community settings, who may potentially benefit from extended or ongoing access to a supportive intervention like the BWW given their persistent mental health needs. On the other hand, a systematic review of predictors of internet interventions for depression and anxiety actually found that a lower rate of baseline symptoms, and less familiarity with psychological treatments was associated with greater program adherence [4]. The low rate of interest in extended access to the BWW may therefore represent a lack of perceived benefit from this type of intervention among more severely symptomatic individuals. Conversely, select users may have benefitted from the first 3 months of access and no longer perceived a need for the intervention. It may be difficult to predict these individuals based on demographic characteristics alone, we found that anxiety level may be important. While some have found that higher co-morbid anxiety is a predictor of drop-out from self-guided web-based interventions for depression [19], studies of face-to-face delivered cognitive behavioral therapy (CBT) have shown that extended access may be more beneficial for those with high anxiety as opposed to only depressive symptoms [20]. The multi-component nature of the BWW with peer support could be particularly appealing for those with higher anxiety levels.

The low observed rates of sustained use we observed are not dissimilar to rates reported across a range of digital mental health applications offering support for mood and anxiety problems [7]. Whereas other evaluations of sustained use or ‘adherence’ with web-based interventions have mainly studied modular style programs such as computerized CBT [7], the BWW is unique in its multi-component structure, making a required ‘dose’ more difficult to define. Defining adequate ‘dose’ has been identified as a particular challenge in evaluating adherence to these types of interventions [6]. The BWW offers a combination of courses and educational material and the peer forum which may appeal differently to users. Some studies have demonstrated better outcomes among users who engage more in cognitive and behavioural tasks, although these differences have not been marked [21]. Conversely, peer communities, particularly if unmoderated, have demonstrated the potential to aggravate depressive symptoms in some individuals who are prone to downward negative cognitive spirals [22].

In this study, we examined use among individuals who opted in for more of the intervention, presuming that they were finding the intervention helpful. Of interest, we found that some participants opted in for more intervention and didn’t use it during the extension period. This may represent a type of safety net or back-up option for some individuals who want access *just in case* they need it or access to other treatment options ends. A pattern of infrequent, intense use of digital health platforms such as patient electronic records has been characterized wherein use of the platform occurs during times of crisis or heightened need with long gaps in between [23]. It has been proposed that relatively brief and/or intermittent use of digital mental health interventions could offer significant benefit on a population level for common mental health needs [7], although this area requires more research. As such, interventions like the BWW are likely to yield highly variable uptake when disseminated to a broad user base. One delivery model would be to make the intervention available at all times to all users who can engage with it whenever they are in need, which for individuals with chronic mental health problems may occur intermittently across various stages of their illness. Alternatively, progressively longer subscriptions or per use fees (paid by any payor) would allow users to determine if and how the intervention works for them and define their own continued use.

## Limitations

Because we concealed the extension opportunity and limited it to survey responders in the main trial, our study population represents a subset of participants only. This was intentional in our study to identify interested users and determine their longer-term use and benefits from the application, however there may have been individuals who did not respond to the main trial survey who were finding the intervention helpful. Additionally, we don’t have any way to know if low interest or decreased use over time reflected a successful use of the materials during the initial exposure period or lack of perceived benefit from the application. Our study population was intentionally heterogeneous and had a high proportion of females reflective of the recruitment settings, which limits interpretation of results for specific diagnostic groups or care settings. Unfortunately, this study had low power to detect differences in the trial outcomes because of the unanticipated dropout from the main trial and the low rate of willingness to opt in for the extension study. We expected a higher rate of participation at the outset and were surprised by the low number of participants we retained. In this case we studied a population who was highly interested in the intervention and/or their mental health recovery which would limit generalizability of findings to other populations, but also highlights the limited uptake of the intervention even among an “interested” population of users. For this reason, recovery is a relevant pan-diagnostic outcome that assesses overall illness recovery and self-management [14]. The potential for self-directed, web-based mental health interventions specifically to support recovery has been discussed [24], however there has been relatively little use of this outcome in trial research. Additionally, we had modest loss to follow-up, not out of keeping with rates as high as 50% reported for trials of web-based mental health interventions [4].

## Conclusions

A high number of individuals given access to a free, web-based multi-component application will use it briefly but not in an ongoing way. Some users may benefit from brief use, while others will use it to support their recovery journey in a continuous way as they find helpful over time. Users with high anxiety may be more likely to request and possibly benefit from longer access periods. A necessary “dose” of this type of intervention (or particular components of the intervention), is difficult to quantify [6] and some may benefit from just the comfort of its availability. Determining which user requires what “dose” and how to optimally implement these and similar web-based self-directed applications interventions at a population level to meet the right individuals in a cost-effective way that considers appropriate payment models, requires careful consideration and is an area that still needs further study.

## Abbreviations

BWW: Big White Wall
CIQ: Community Integration Questionnaire
GAD-7: Generalized Anxiety Disorder Questionnaire – 7 item
IQR: Interquartile range
PHQ-9: Patient Health Questionnaire – 9 item
RAS-r: Recovery Assessment Scale – revised
VAS: Visual Analog Scale
